# The regulation of cognitive enhancement devices: extending the medical model

**DOI:** 10.1093/jlb/lst003

**Published:** 2014-02-25

**Authors:** Hannah Maslen, Thomas Douglas, Roi Cohen Kadosh, Neil Levy, Julian Savulescu

**Affiliations:** 1 Research Fellow in Ethics, Oxford Martin School, University of Oxford and Junior Research Fellow, New College, University of Oxford; 2 Senior Research Fellow, Oxford Uehiro Centre for Practical Ethics, University of Oxford and Golding Fellow, Brasenose College, University of Oxford; 3 Wellcome Trust Research Career Development Fellow and University Research Lecturer, Department of Experimental Psychology, University of Oxford; 4 Australian Research Council Future Fellow, Florey Institute of Neuroscience and Mental Health; 5 Uehiro Chair in Practical Ethics, Oxford Uehiro Centre, University of Oxford, Suite 8, Littlegate House, 16/17, St. Ebbe's Street, Oxford, OX1 1PT, UK

**Keywords:** cognitive enhancement devices, Medicines and Healthcare Products Regulatory Agency: medical devices, regulation, transcranial direct current stimulation

## Abstract

This article presents a model for regulating cognitive enhancement devices (CEDs). Recently, it has become very easy for individuals to purchase devices which directly modulate brain function. For example, transcranial direct current stimulators are increasingly being produced and marketed online as devices for cognitive enhancement. Despite posing risks in a similar way to medical devices, devices that do not make any therapeutic claims do not have to meet anything more than basic product safety standards. We present the case for extending existing medical device legislation to cover CEDs. Medical devices and CEDs operate by the same or similar mechanisms and pose the same or similar risks. This fact coupled with the arbitrariness of the line between treatment and enhancement count in favour of regulating these devices in the same way. In arguing for this regulatory model, the paper highlights potential challenges to its implementation, and suggests solutions.

## The regulation of cognitive enhancement devices: extending the medical model

A major challenge facing EU regulatory bodies is how to regulate devices intended for cognitive enhancement. These are devices like brain stimulators, which modify the electrical activity of the brain, and sometimes its physical structure. Whilst there have been a number of calls from groups such as the British Medical Association (2007)^1^ for more policy debate on enhancement technologies, few specific recommendations have been made. Further, the debate that has taken place has focused predominately on pharmaceutical cognitive enhancers—drugs developed for medical conditions that are being used off-label to improve things like concentration, impulse control and memory in ‘healthy’ individuals. Non-pharmaceutical devices intended for enhancement have received little regulatory attention until very recently.

The regulation of technologies can occur at many points from the research and innovation stages, through placing a technology on the market, to the use of the technology by private individuals. Whilst the EU has a clear regulatory framework for medical devices, it is yet to develop anything comparable for CEDs. CEDs, though similar to medical devices in their modes of action, are sold and used not to treat disease, but to augment typical cognitive capacities for purposes ranging from accelerating academic learning to augmenting performance in online gaming. The regulatory gap is particularly concerning given that these potentially risky devices are being bought and used by individuals with little knowledge and training, and in the absence of rigorous regulatory safeguards. For example, Transcranial Direct Current Stimulators (tDCS) and neurofeedback devices are currently marketed online as CEDs without the particular models on sale undergoing comprehensive clinical evaluation.

In a short commentary paper and an even shorter correspondence piece we have recently proposed that CEDs should be regulated in the same way as medical devices.^2^^,^^3^ However, a comprehensive defense of this position and an examination of its legislative ramifications have yet to be undertaken. This paper is the first to make a detailed and sustained case for regulating CEDs on the same model as medical devices, and to identify and scrutinize the legislation pertinent to the implementation of this model. Having set out the possible regulatory options, we argue that the existing medical device legislation should be amended so that it also regulates which CEDs are placed on the market. In setting out and defending our preferred regulatory model, we highlight potential challenges to its implementation, and suggest solutions.^4^

## WHAT ARE COGNITIVE ENHANCEMENT DEVICES?

A cognitive enhancement device is a piece of equipment or combination of pieces of equipment that is sold and used to affect the functioning of the brain such that it performs better in at least one cognitive domain (eg memory, attention, learning, facial recognition). Where the device is also (and perhaps even simultaneously) sold and used to treat a disease, it might also qualify as a medical device. Perhaps the most widely available CEDs are brain stimulation devices—devices that use electrical current to modulate specific areas of brain activity. Other devices encompassed by this definition include equipment used for neurofeedback training—a process by which individuals can learn to exert control over certain mental states through real-time monitoring of their own brain activity. Below we describe two CEDs that are increasingly being marketed online without being held to anything more than basic product safety requirements.

### Transcranial direct current stimulators

Transcranial direct current stimulation is the most widely marketed kind of brain stimulation device for cognitive enhancement. Recent reports have emphasized how easy it is for individuals to purchase these devices online.^5^ There are many websites through which it is possible to purchase a device or components for a device.^6^ Although no sales figures have been published, the newest addition to the tDCS device market is currently listed as being sold out.^7^ Further, some non-medical clinics are offering tDCS as an ‘experimental therapy’ to help with ‘anxiety and mood; cognitive performance (learning, memory, concentration, focus); stroke; migraine’.^8^

TDCS is a non-invasive technique in which a device sends a small direct current between electrodes placed on the scalp to stimulate or inhibit spontaneous neuronal activity. Weak electrical currents, usually in the order of 1–2 mA, are applied. The electrodes, most frequently at the size of 25–35 cm^2^, are placed on the scalp above the area that the experimenter is interested in affecting. When the current is applied constantly over a short duration (∼10–20 min) it passes painlessly through the scalp and skull and alters spontaneous neural activity.^9^ To date, a number of clinical studies have reported some promising effects of tDCS when treating patients with depression, chronic pain, schizophrenia, dementia, Parkinson's disease and cerebral stroke.^10^ However, tDCS has also been used in healthy individuals, with studies from different labs showing the potential of tDCS to improve cognitive abilities including working memory, attention, language, mathematics, and decision-making.^11^

There are risks and other concerns associated with a tDCS device that is structured or functioning suboptimally.^12^ The electrodes must be positioned correctly in order to produce reliable effects.^13^ This means that devices must be constructed so that the user is easily able to position the electrodes to produce the desired effect. Ensuring the correct placement of electrodes may be made more difficult when the user is left-handed due to handedness-related differences in brain organization.^14^ Devices that enable the polarity of the stimulation to be reversed pose risks as this can impair brain function: Reversing the polarity of the electrodes may not only be ineffective in producing enhancement but may also result in impaired neuronal function.^15^ The strength and duration of stimulation the device delivers will affect how safe it is to use. Stimulation that is too strong or stimulation that exceeds the optimum duration may be damaging, so devices that have the capacity for delivering strong stimulation or allowing extended continuous use will pose risks to users.^16^

There are also risks and safety concerns associated with the intentional or unintentional misuse of tDCS devices.^17^ If the user is taking any medication or other psychoactive substances, these might interact with the stimulation effects resulting in desirable or undesirable outcomes: Given the wide variety and availability of substances with the potential to interact with tDCS, the lack of knowledge of these effects increases the risks posed to those purchasing devices for enhancement. In relation to prescription medication, studies have shown that a person's baseline cortical excitability can differ if they are taking certain drugs, including benzodiazepines, anticonvulsants, antidepressants, and others.^18^ Further, people who present with neuropsychiatric disorders such as depression, schizophrenia or migraines also show differences in baseline excitability compared to healthy individuals.^19^ These differences mean that the same amount of current is likely to have non-uniform effects in users taking different psychoactive substances and with different neurological conditions.

Effects of tDCS may be unintended and long lasting: While the majority of studies have demonstrated only short-term changes in the brain, others have reported effects lasting for at least six months.^20^ Particularly, if suboptimal tDCS devices are being used, or devices are being used incorrectly, there is a risk that undesirable changes to the user's brain and its functioning may become difficult to reverse. Even where tDCS is used correctly and achieves the desired effect, there may be unintended effects on neurobiology. An example of this phenomenon can be seen in studies in which tDCS stimulation of the posterior parietal cortex enhanced numerical competence, but when automaticity for the learned material was measured, deficit was noted: whilst stimulation to the posterior parietal cortex facilitated faster learning, it simultaneously impaired the automatic processing of the learned material.^21^

A further concern is that the use of a tDCS device on a developing brain—particularly the prefrontal cortex—might lead to atypical brain development.^22^ Like other types of atypical experience during sensitive periods, the stimulation of the wrong brain area might induce abnormal patterns of brain activity in this brain region and interconnected areas, and increase metabolic consumption in brain areas that are irrelevant to the specific psychological function.^23^

### Neurofeedback equipment

Neurofeedback is a type of biofeedback that uses realtime displays of brain activity based on neuroimaging, often with the goal of enabling the person to regulate his or her brainwave activity. This is achieved through a process of operant conditioning. Neurofeedback training typically involves placing electrodes on the person's scalp to measure the electrical patterns emanating from her brain. Connected to a computer, the person receives instantaneous auditory and visual feedback about her brainwave activity. Having awareness of her brainwave patterns enables the person to learn to reinforce or suppress different patterns of activity. Particular patterns are associated with inwardly focused attention, others with outwardly focused alertness and others still with relaxation, daydreaming and sleep.^24^ Depending on the desired state, neurofeedback can be used to cultivate different patterns. With repeated feedback training and practice, desirable brainwave patterns can usually be retrained in most people. ^25^ As with tDCS devices, there are many websites selling neurofeedback devices and equipment.^26^ There are also non-medical clinics offering neurofeedback to improve functioning in ‘emotional self-regulation (improved mood, reduced anxiety, anger); stress management; focus, concentration, attention; cognitive performance, including memory; energy and motivation (reduced fatigue); better sleep’.^27^

In the clinical domain, neurofeedback has been used to help patients with attention deficit hyperactivity disorder, epilepsy, autism, and insomnia.^28^ However, as with tDCS, neurofeedback has also been used in healthy individuals to enhance attention, memory, microsurgical skills, intelligence and well-being. Further studies have shown that neurofeedback can be used to enhance musical creativity in children, and to enhance the dancing and acting performance of adults.^29^^,^^30^^,^^31^

Although the risks associated with neurofeedback are not of the same gravity as with tDCS, it is not completely risk-free. Mild side effects such as fatigue, anxiety and irritability can sometimes occur during neurofeedback training.^32^ In some people, neurofeedback training can also lead to headaches, muscle twitches, tics, mental fogginess, and sleep disturbance.^33^ It is thought that some people are particularly vulnerable to over-training, resulting in a transient decrease in cognitive functioning and other side effects.^34^ Further, unless the training is carefully tailored to the individual, there will be a risk that it will be ineffective or even produce an adverse reaction: Due to the heterogeneity in the brainwave activity, training must be individualized, and research is increasingly showing that different treatment protocols have differential effects on the brain.^35^ The potential for adverse effects is perhaps made more worrying by evidence that neurofeedback training can lead to microstructural changes in white and gray matter.^36^

## WHY ARE COGNITIVE ENHANCEMENT DEVICES NOT TIGHTLY REGULATED IN EUROPE?

In order for a regulatory body to set standards for a particular technology, it first has to identify the technology as something that requires regulation. One problem with current EU legislation is that products intended for enhancement are not identified by any of the existing directives other than those covering general product safety - the General Product Safety Directive (GPSD).^37^ The GPSD, however, only sets general requirements. According to Article 2(b) of the GPSD, a ‘safe product’ is:

any product which, under normal or reasonably foreseeable conditions of use including duration and, where applicable, putting into service, installation and maintenance requirements, does not present any risk or only the minimum risks compatible with the product's use, considered to be acceptable and consistent with a high level of protection for the safety and health of persons, taking into account the following points in particular:

- the characteristics of the product, including its composition, packaging, instructions for assembly and, where applicable, for installation and maintenance;
- the effect on other products, where it is reasonably foreseeable that it will be used with other products;
- the presentation of the product, the labeling, any warnings and instructions for its use and disposal and any other indication or information regarding the product;
- the categories of consumers at risk when using the product, in particular children and the elderly.

Crucially, whereas many medical devices must undergo rigorous clinical assessment before being approved for placement on the market, the GPSD does not make provision for pre-market assessment. CEDs, despite often raising safety and effectiveness concerns comparable to those raised by medical devices, are not covered by the Medical Devices Directive (MDD)^38^ because the definition the directive employs excludes them. The current definition of a medical device specifies that the device must be intended by the manufacturer to be used for diagnostic and/or therapeutic purposes. Since CEDs are neither diagnostic nor therapeutic, they are not identified as devices for medical regulation. Article 1(2) (a) of the MDD defines a medical device as^39^:

Any instrument, apparatus, appliance, software, material or other article, whether used alone or in combination, including the software intended by its manufacturer to be used specifically for diagnostic and/or therapeutic purposes and necessary for its proper application, intended by the manufacturer to be used for human beings for the purpose of:

- diagnosis, prevention, monitoring, treatment or alleviation of disease,
- diagnosis, monitoring, treatment, alleviation of or compensation for an injury or handicap,
- investigation, replacement or modification of the anatomy or of a physiological process,
- control of conception,

and which does not achieve its principal intended action in or on the human body by pharmacological, immunological or metabolic means, but which may be assisted in its function by such means.

It might be argued that the definition in its current form in fact *does* cover CEDs: devices used for tDCS or neurofeedback modify physiological processes in the brain, as per the third indent of the definition. However, if this criterion were considered individually sufficient, the definition would then problematically extend to anything that alters the brain: books, DVDs and computer games would arguably become medical devices, as interacting with them to some extent modifies neuronal connections.

Further, the consensus amongst the European Commission, Member States and stakeholders is, reportedly, that having a specific diagnostic and/or therapeutic purpose is the primary criterion for a medical device, and that the purpose of ‘investigation, replacement or modification of the anatomy or of a physiological process’ is amongst the secondary criteria once a general medical purpose has been recognized. A working document published by the European Commission during the recent consultation on the MDD explains:^40^

It is currently not clear whether implantable or other invasive products for which the manufacturer does not claim a medical purpose, but eg an aesthetic or cosmetic purpose, are covered by the AIMDD [Active Implantable Medical Device Directive] or MDD or not. Some argue that the third indent of the ‘medical device’ definition in Article 1(2)(a) of the MDD covers any device which pursues the purpose of ‘investigation, replacement or modification of the anatomy or of a physiological process’, regardless of whether the manufacturer attributes to it a medical or a non-medical (eg aesthetic) purpose. However, according to the prevailing interpretation of the Commission, Member States and stakeholders, a device falls within the definition of a medical device when it pursues a medical purpose. The question is currently pending before the European Court of Justice for a preliminary ruling.

The result is that CEDs, despite modifying physiological functions, are not identified by the definition as devices for regulation. ^41^

## POSSIBLE APPROACHES TO CED REGULATION

### What proposals for CED regulation have there been?

The present lack of a rigorous regulatory process for CEDs—and for enhancement technologies more generally—has motivated large-scale working groups to consider the ethical and social implications of the increasing production and use of these technologies. For example, the British Medical Association published a report in 2007 on the ethical aspects of cognitive enhancement; the European Commission funded a 7th framework program on *Ethics in Public Policy Making: The Case of Human Enhancement (EPOCH);*^42^ the Academy of Medical Sciences, in collaboration with the British Academy, the Royal Academy of Engineering and the Royal Society, published a report based on their workshop investigating *Human Enhancement and the Future of Work*^43^.

Notwithstanding the various important outputs of these and similar projects, there has been sparse overt guidance to lawmakers and regulatory bodies on the regulation of cognitive enhancement technologies. As summarized by Outram and Racine (2011),^44^ the report published by the British Medical Association (BMA)^45^ places emphasis on public debate in advance of making recommendations. Whilst it outlines the possible regulatory approaches and discusses their implications, it does not argue for the adoption of any particular course of action. The express aim of the BMA report is to facilitate informed debate amongst doctors, scientists, policymakers, and members of the public about the future development and use of cognitive enhancements. The BMA states that it ‘does not have policy or recommendations to put forward on these issues but would welcome informed public debate about how, as a society, we should respond to these developments’.^46^

The aim of the EPOCH project was to broaden and deepen knowledge of the role of ethics in the governance of science and technology, focusing on ethical aspects of new and emerging bio-, neuro- and nano-technologies and specifically related to the topic of human enhancement. Although regulatory challenges were a focus of the project, the central aim was to generate new insights into the role of ethical expertise in European policymaking on science and technology, coherent with national and other European projects. Although the EPOCH Project is yet to comprehensively publish its findings and recommendations, the development of a regulatory model for enhancement technologies was not amongst the stated aims of the project.

The recent report from the joint academies had a narrow focus on human enhancement in the workplace. The report suggests that the greatest immediate challenges for regulators and other policy-makers will arise from the use of drugs, brain stimulation, and digital devices that enhance cognition and concludes that dialogue with potential users and the wider stakeholder community, as well as studies and commissioned research, will be required to balance the risks and benefits of these technologies in the future workplace. The report does go some way towards suggesting particular regulatory approaches, but these recommendations are specific to employment contexts. As the report notes, ‘in many ways, work represents a unique context, within which a cautionary regulatory approach is desirable, with the primary objective of protecting employees’.^47^ We should not assume that the regulatory approach appropriate for work contexts will also be appropriate for other contexts.

More recently, attention has been paid in particular to the lack of regulation for tDCS devices used outside the clinical setting. Emphasizing that tDCS is not without safety concerns, Fitz and Reiner^48^ call on regulators, scientists and the tDCS DIY community to develop policy proposals that ensure public safety while supporting DIY innovation. To our knowledge, only the Nuffield Council on Bioethics^49^ has outlined a model for the regulation of neurotechnologies used for enhancement. In concord with the model we develop, The Nuffield Council proposes that neurotechnologies such as tDCS should be regulated in the same way as medical devices. However, an in depth discussion of the existing legislation and exploration of how the model used for medical devices (henceforth the medical model) could be implemented has not yet been undertaken. The remainder of this paper will examine the possible regulatory options and argue that the best approach is one in which medium and high-risk CEDs are regulated in the same way as medical devices, with low risk devices held to a less stringent standard. We explore the implications of this model within the European context and make recommendations for how it should be implemented.

### The eight regulatory options

The possibilities for the regulation of CEDs can be identified according to (1) the regulatory instruments that could be employed, and (2) the stringency of the standards CEDs could be required to meet. CEDs could be regulated under the same legislation as medical devices (the MDD), they could be regulated by a new regulatory body/under new legislation specifically for CEDs, the status quo, in which CEDs fall only under general product safety regulations, could be maintained, or, finally, CEDs could be prohibited entirely. Adopting either of the first two options would allow CEDs to be held to a higher regulatory standard than medical devices, to the same standard or to a lower standard. This therefore generates eight options to consider:

- CEDs could be regulated via a new process specifically for CEDs, to:
  1) …a higher regulatory standard than medical devices,
  2) …a lower regulatory standard than medical devices, or
  3) …the same regulatory standard as medical devices.
- CEDs could be regulated under the same legislation as medical devices, to:
  4) …a higher regulatory standard than medical devices,
  5) …a lower regulatory standard than medical devices, or
  6) …the same regulatory standard as medical devices.
  7) The status quo could be maintained.
  8) CEDs could be prohibited.

Arguments can be made to reject options 7) and 8) at the outset. For the reasons stated above, maintaining the *status quo* is not a defensible option. The devices currently marketed for cognitive enhancement are most often devices that are also being tested in clinical research trials, with the hope that ultimately they will be used to treat patients. For example: to date, a number of clinical studies have reported some promising effects of tDCS when treating patients with depression, chronic pain, schizophrenia, dementia, Parkinson's disease and cerebral stroke.^50^ Whether used in research, for treatment or for enhancement, the devices modify brain activity via similar mechanisms and with similar physiological effects. They can thus be expected to impose similar risks; and there seems little reason to suppose that CEDs offer greater benefits. Given these facts, the careful regulation of the same or similar devices in one context but not in others appears arbitrary. At the opposite end of the spectrum of policy options, there does not seem to be a convincing argument for a complete prohibition of CEDs. Although devices will present some risks, these are not greater than the risks posed by many medical devices that are considered safe enough to be placed on the market.

Some authors have argued that using biotechnologies for cognitive enhancement, or indeed to enhance other human capacities, is morally problematic for reasons other than risk, for example because it invariably expresses an objectionable desire to ‘master’ the human body and mind, or because it can be expected to have net harmful social consequences.^51^ Such arguments could be taken to support a universal prohibition on CEDs, among other enhancement technologies. However, these arguments have been strongly contested.^52^ Moreover, even if CED use is always morally problematic, this may not justify legal or regulatory prohibition. A concern to protect individual autonomy would militate against such a prohibition, and it might also be argued that CEDs ought to be permitted in order to help forestall unregulated illicit use. As noted by Cohen Kadosh et al,^53^ devices can be built from off-the-shelf components: it is preferable that, if individuals choose to pursue enhancement, they purchase devices that are held to a strict level of safety, appropriate for the particular use intended. Finally, while other forms of biomedical enhancement, such as cosmetic surgery, remain permitted, there is an argument from consistency for allowing the use of CEDs as well.

The remaining two groups of options require more detailed discussion. CEDs could be regulated separately from medical devices by a new regulatory process. Alternatively, CEDs could be regulated in the same way as therapeutic medical devices, for example by extending the definition of a medical device to include devices with enhancement purposes. These two options will be examined in turn, with the authors arguing in favor of the latter approach. As above, the focus remains on *European* and in some cases British regulation, though we believe many of our arguments could be extended to other contexts.

### Option one: establish a new regulatory process of CEDs

Whether or not CEDs are regulated via the same process as medical devices, they could be held to the same regulatory standard, to a lower standard, or to a higher standard. Thus, a desire to hold CEDs to a lower or higher regulatory standard than medical devices need not militate in favor of establishing a new regulatory process for CEDs. Rather, the most obvious argument for establishing a new regulatory process would maintain that CEDs are categorically different from medical devices, raise qualitatively different regulatory issues, or ought to be held to standards so high that their regulation must proceed in a significantly different way (bearing in mind that there is already provision for variation of standards within the MDD). As a way of regulating CEDs separately from medical devices (but along with cognitive enhancing pharmaceuticals), the BMA suggests as a possibility ‘the establishment of a new regulatory body to approve the use of particular techniques and to issue guidance for their use—the Regulatory Authority for Cognitive Enhancements (RACE) perhaps?’^54^

However, there are practical arguments against establishing a new regulatory process, particularly if this were to involve the creation of new government agencies. Indeed, the BMA point out the EU's problem with the proliferation of regulatory bodies^55^:

It has been argued that the UK suffers from regulatory overload and that state intervention in individual choices made by patients, in consultation with their doctors, should be kept to an absolute minimum. The establishment of a statutory regulatory body is expensive, bureaucratic and involves considerable work and time from those regulated.

This practical concern, however, is not the only argument against regulating CEDs independently from medical devices: there are also strong theoretical reasons to resist a separate regulatory process. First, CEDs are not categorically different from medical devices; in fact, the very same device may be used both for therapeutic and enhancement purposes, in some cases using similar parameters.^56^ CEDs, as devices that modify brain function to improve cognitive performance are, in important respects, the same *sorts* of devices that the MDD covers: they intervene to modify physiological processes and present varying degrees of physiological risks and side effects. Whilst in some cases there is no categorical distinction to be made between CEDs and medical devices, it is true that the *purpose* of CEDs is enhancement and not therapy. However, the proposed revision of the MDD to cover (principally cosmetic) devices without a medical purpose sets a precedent for non-therapeutic devices to be regulated in the same way as medical devices.^57^ It could even be argued that aiming to improve cognitive function is closer to our traditional understanding of medical purpose than is aesthetic enhancement, and it is certainly not further from therapy than is cosmetic surgery. If cosmetic devices are not out of place within the MDD, then neither are CEDs.

There is also a philosophical reason to place CEDs within current medical regulatory regimes. Many philosophers have denied that there is a morally relevant difference between treatment and enhancement.^58^ Both therapy and enhancement aim to improve a human being's biology and/or psychology. The two most important ethical considerations in regulating such interventions are the risks that are involved and considerations of distributive justice when such interventions are publicly funded. It is plausible that treatments raise these concerns in similar ways to enhancements. Thus, the critical issue in the evaluation of any new technology, whether for treatment or enhancement, is to ascertain the likely benefits (including in terms of increments in wellbeing) and the risks. The balance of benefit over risk is only one determinant in deciding whether interventions should be admitted to the market place, restricted or publicly funded.

Finally, the potential worry that some CEDs should be held to a higher standard than medical devices does not preclude regulation under the MDD. In fact, different medical devices are already held to different standards within the directive. Further, in the UK, the Medicines and Healthcare Products Regulatory Agency (MHRA) has proposed that implantable or other invasive products without a medical purpose be regulated through the same process as medical devices, but held to a different (more stringent) standard than many of the regular medical devices, requiring that they present zero or minimal risk. There is therefore the possibility of setting an appropriate standard for CEDs within the MDD (see below for discussion of weighing the risks and benefits of CEDs).

The European Commission, in its discussion of the policy options for extending the medical devices definition to cover some implantable or other invasive products without a medical purpose, endorses arguments similar to those we have advanced above. While the Commission does think there may be merit in considering products with a medical purpose separately from those without such a purpose, it also sees theoretical value in retaining a homogenous definition and assessment framework, and practical value in having legislation that can more easily be extended in the future. Ultimately, the Commission considers the costs of separate legislation to be too high:^59^

The negative impact of a separate legislation would be that manufacturers which produce same or similar products with and without a medical purpose (eg corrective and non-corrective contact lenses without medical purpose) would be subject to two different product-related legislations which, in particular for [small and medium sized enterprises], would be more burdensome and increase compliance costs.

Moreover, it would not appear logical to submit products which have the same features and the same risk profile to different requirements. In addition, experiences gained under one legislation (eg vigilance reporting) could not be easily taken into account for regulatory purposes for products subject to another legislation.

These points echo our practical points as well as our claim that CEDs are not categorically different, even if they can be used for different purposes. In fact, unlike aesthetic products, which may have no similarities to medical devices, existing CEDs are all very similar if not identical to devices recognized as medical devices.

### Option two: include CEDs within the regulatory process for medical devices

The second option—and the regulatory model we wish to present and defend—would be to accommodate CEDs within the existing regulatory process for medical devices. We assume that this would be achieved in Europe by revising the MDD to include CEDs within the category ‘medical devices’. In revising the MDD, two possibilities present themselves: either the core definition of a medical device might be revised so that the potential purposes attributed to them include (or do not exclude) enhancement, or an ancillary ‘positive list’ of CEDs might be drawn up to supplement the existing definition. This latter option has been proposed by the MHRA as the preferred method for extending the directive to cover some implantable or other invasive products used for a non-medical purpose. The MHRA's proposal does not cover CEDs as it is intended (and formulated) only to bring certain devices with an *aesthetic* or *cosmetic* purpose within the remit of the MDD.

To adequately assess the optimal approach for CEDs, various conceptual and practical questions need to be considered. The first conceptual question is whether, by amending the legal definition of a medical device, legislation explicitly intends to alter how we understand the term ‘medical device’, or whether the amendment is based merely on the view that the same regulatory instruments should apply to both medical devices and CEDs. If the intention is in part to alter how ‘medical device’ is understood—what it means for a device to be a medical device—then there would be a strong case for altering the core definition of a medical device so that the potential purposes attributed to them include (or do not exclude) enhancement. If, instead, the aim is merely to ensure that the directive (and its regulatory apparatus) is applied to CEDs, then it may be preferable to include an ancillary ‘positive list’ of CEDs that are to be included within the remit of the MDD, whilst not themselves being classed as medical devices.

The second conceptual question is whether the set of devices the definition is extended to cover should be determined by the way in which the device interacts with the body or by the purpose for which it is used. The existing core definition of a medical device focuses on purposes, and it might be difficult to amend it to accommodate an additional class of devices that are defined in part according to their mode of interaction with the body. On the other hand, an ancillary list could be generated based either on the type of interaction with the body—eg brain stimulation devices—or by identifying the particular purpose—cognitive enhancement. The MHRA's proposal for inclusion of a positive list of implantable or other invasive products without a medical purpose takes the first of these two approaches: the devices share the feature that they are implantable or invasive, and the purpose of cosmetic enhancement is thus not the categorizing factor.

The practical question to be considered alongside these conceptual issues is how the regulators are best able to ‘keep control’ of which technologies the MDD covers. A positive list allows for better control. Of course, such a list would need to be regularly updated. A benefit of amending the core definition of a medical device is that new CEDs would be held to the required standards from the moment of their emergence on the market (indeed, their emergence on the market would be dependent on meeting the standards). A positive list that was reactive to CEDs already in use creates the risk that untested devices might be used for some time before being subject to regulation.

Whether a purpose-based or device-based definition is used, it is important that such an approach is not overly inclusive, for example including educational training software. We suggest that a further necessary condition be included in any regulatory framework: that regulation is only appropriate on grounds of potential risk. That is, whether regulation is purpose- or device-based, it should be risk-oriented. Only technologies which involve more than minimal risk should fall under regulatory purview.

We return to these issues later when offering our preferred approach. At this point, however, we hope to have convinced the reader that an amendment of the MDD of some sort is needed. The principle justifications are:

- CEDs and medical devices are similarly-acting technologies which can pose similar risks—there is thus no relevant distinction between devices used for treatment and enhancement in terms of mechanism or risk.
- There is no morally relevant distinction between the purposes of treatment and enhancement. Both therapy and enhancement aim to improve a human being's biology and/or psychology.
- Parsimony in regulation is always preferred where possible.
- The implantable and other invasive products without a medical purpose that are already included on a positive list primarily have cosmetic purposes—this is arguably further from medical purpose than is enhancement purpose and thus sets a precedent.

Amending the definition presents two significant challenges which would need to be resolved: (1) how the purpose of a device is identified; (2) how benefits are quantified and (3) how any risks and side effects should be weighed against the benefits of enhancement, essentially setting the stringency of the regulatory requirements for CEDs. We explore these challenges in turn.

## AMENDING THE MEDICAL DEVICES DIRECTIVE

### Challenge one: identifying purpose (enhancement is often a secondary purpose)

If the definition of a medical device were to be amended to include (or cease to exclude) CEDs by including devices with an *enhancing* purpose, thought would have to be given to how the enhancing purpose of a device is identified. The current wording of the directive provides that medical devices are devices intended by their manufacturer to be used specifically for diagnostic and/or therapeutic purposes. In some cases, this means that the very same device is identified for regulation as a medical device when marketed as such, but not when it is marketed ‘off-label’ as a cognitive enhancement device.

Crucially, the wording of the definition suggests that what comes to be regulated under the directive depends on the explicit claims manufacturers make about their products. A guidance document published by the European Commission elaborates on how this purpose is identified:^60^

Medical devices are defined as articles which are intended to be used for a medical purpose. The medical purpose is assigned to a product by the manufacturer. The manufacturer determines through the label, the instruction for use and the promotional material related to a given device its specific medical purpose.

Given that the medical purpose of a device is identified in this way, if the definition were to be extended to include (or to cease to exclude) the enhancing purposes of various devices, enhancement purposes might be derived from the manufacturer's labels and instructions, and so forth. However, there might be a difficulty in identifying purpose when a device is marketed for both therapy and enhancement; one option, in such cases, would be to treat the device as *both* a CED and a therapeutic medical device.

It might be possible to avoid these difficulties by identifying and including CEDs not on the basis of their purpose, but rather by their mode of interaction with the body. For example, all devices that electrically stimulate the brain might be classed as medical devices, regardless of the purpose of that stimulation. However, there might still be reasons to identify the purpose of brain stimulation devices. For example, adjudicating between purposes might be significant for determining the level of safety required: if the benefits of a device used for enhancement would typically be less than the benefits of the same device used for therapy, then it might be appropriate to require a lower level of risk in order for the device to be approved for its enhancement purpose. How to weigh risks against the benefits of enhancement is considered in the next section.

### Challenge two: risk-benefit assessment (how measurable are the risks and benefits of enhancement?)

If CEDs were regulated within the existing definition, they would be subject to the general requirements emphasizing safety and effectiveness, requiring risks to be weighed against benefits. Whilst the risks and side effects of CEDs could be assessed in a similar way to the risks and side effects associated with medical devices, it is less clear how the *benefits* of CEDs should be measured. Should a similar measure of *effectiveness* be used to determine the benefit an enhancement device confers? Speaking against adopting an ‘effectiveness’ approach, it could be argued that unlike medical devices—which either succeed or fail in improving or maintaining health to a measurable degree—CEDs confer benefits that are more subjective. Parallels might be drawn with the difficulty of assessing the benefits of cosmetic enhancements: a nose might be made smaller or straighter in a way that we can measure, but how *beneficial* this is will vary from person to person that is not captured by an assessment of *effectiveness*.^61^

It is certainly possible to measure the size of any improvement to cognitive performance. For example, the improvement in learning speed or capacity of an individual using tDCS will be something determinable through laboratory tests that assess the respective skill acquisition. However, whilst we can measure the *size* of improvements to cognitive functions (effectiveness), it could be argued that the *value* of enhancement is something that varies between people to a greater extent than the value usually attached to health. Intuitively, the degree of subjectivity in the value of cognitive enhancement sits somewhere between the (arguably) more objective value of health and the more subjective value of the physical traits produced by cosmetic surgery. The value of these traits is arguably highly subjective both because there are differences of opinion concerning what *is* aesthetically appealing and because the value of possessing aesthetically appealing features itself depends on the psychology of the individual. Thus, a significant issue to resolve when extending the MDD to cover CEDs is how the benefits of the devices are to be estimated and weighed against any risks or side effects. It appears to be the view of the European Commission that, as measurable benefits fall, less risk should be tolerated. We derive this understanding from the basic requirements pertaining to the safety and performance of medical devices:^62^

Devices shall achieve the performance intended by the manufacturer and be designed and manufactured in such a way that, during normal conditions of use, they are suitable for their intended purpose, taking into account the generally acknowledged state of the art. They shall not compromise the clinical condition or the safety of patients, or the safety and health of users or, where applicable, other persons, provided that any risks which may be associated with their use constitute acceptable risks when weighed against the benefits to the patient and are compatible with a high level of protection of health and safety.

Further instruction on what should be fed into the risk/benefit assessment is found in the European Commission's guide to clinical evaluation, for manufacturers and Notified Bodies.^63^ There it is stated that combined clinical data must show that:

[A]ny risks associated with the use of the device are acceptable when weighed against the benefits to the patient. Such considerations should take into account the number of patients exposed to the device, the type and adequacy of patient monitoring, the number and severity of adverse events, the adequacy of the estimation of associated risk for each identified hazard, the severity and natural history of the condition being diagnosed or treated. The availability of alternative diagnostic modalities or treatments and current standard of care should also be taken into consideration.

The emphasis on an assessment of the severity and natural history of the condition being diagnosed or treated implies that the European Commission holds the view that the more severe the condition, the greater the benefits that can be expected of the device, and thus, the higher the level of risk that can be tolerated. This approach is similar to that used by the UK Human Fertilization and Embryology Authority in regulating the use of preimplantation genetic diagnosis for non-medical purposes such as sex selection. The corresponding idea—that the less severe the condition, the lower the tolerable level of risk—suggests that, as devices move closer to enhancement than treatment, the number and/or magnitude of the risks tolerated will decrease. It seems that the European Commission took such an approach when proposing the amendment for implantable and other invasive devices without a medical purpose. Qualifying the general requirements pertaining to performance and safety, it is suggested:^64^

For devices listed in Annex XV for which the manufacturer does not claim a medical purpose, the general requirements set out in Sections 1 and 5 shall be understood that the device, when used under the conditions and for the purposes intended, shall not present any risk or only the minimum acceptable risks related to the product's use which is consistent with a high level of protection for the safety and health of persons.

Echoing this, the MHRA's consultation document explains:^65^

Weighing up the risks and benefits of a product which does not have a medical purpose is different than for medical devices. Therefore Annex I, which sets out the safety and performance requirements of devices, requires manufacturers of implantable or invasive products without a medical purpose to ensure that these products present either no or the minimum acceptable risk which is consistent with a high level of protection for the safety and health of persons.

This qualification has the result that devices without a medical purpose—even when they have the same risk-profile as analogous devices with a medical purpose—will be held to more stringent standards than devices with a medical purpose: requiring zero or minimal acceptable risk (where this is defined independently of benefits) is more cautious than requiring that risks are acceptable when weighed against the benefits to the patient. Possibly a formulation that omits consideration of benefits was adopted because it was considered that the devices for which no medical purpose is claimed do not confer (relevant, measurable) benefits on their users. The purposes of the devices included in the ‘positive list’ are principally cosmetic, and as suggested above, it might be thought that, as cosmetic benefits are subjective, they cannot be relevant to a risk/benefit assessment.

Whilst not explicitly claiming that cosmetic benefits are unquantifiable, a related sentiment is found in one of the consultation responses from the German trade association representing the National and International Companies of Contact Lens (and Lens Care) Manufacturers:^66^

Since non-corrective lenses for cosmetic/aesthetic purpose, have the same risk profile but no medical benefit, the risk-benefit principle cannot apply as for regular medical devices. Therefore for quasi-medical devices, the principle of keeping the risk as low as reasonably possible (ALARP) should apply. Quasi-medical devices should be classified the same way as medical devices under the principles of Annex IX of Directive 93/42/EC to ensure a conformity assessment route that is equivalent to the risk associated with the device.

The suggestion seems to be that, if a product (such as a non-corrective lens) does not confer *medical* benefit, then it would be impossible or inappropriate to apply the risk-benefit principle: it ‘cannot’ apply. The corresponding justifications for this would be either that non-medical benefits are too difficult to measure (risk-benefit assessment is impossible) or that they are just not relevant to the regulatory assessment of quasi-medical devices (risk-benefit assessment is inappropriate). Perhaps it is considered that regulatory protection of the consumer against exposure to risks and side effects can only be compromised when there is clear evidence that doing so is likely to improve a suspected or diagnosed medical condition. Such a view would hold that no other *sorts* of benefits are important enough to justify such a compromise, even if it were accepted that other sorts of benefits could be identified.

The principle of ‘keeping the risk as low as reasonably possible’ (where ‘reasonably’ is understood without reference to the benefits) endorsed in the above quotation would operate to set the maximum risk that an approved device could pose. With this principle operating alone—as is proposed for invasive or other implantable products without a medical purpose—the result is that *any* device posing a risk greater than the maximum would not be approved for placement on the market. This approach forgoes any risk-benefit assessment in favor of pure consumer protection. In comparison, when the risk-benefit principle is applied, two devices posing the same level of risk might receive different verdicts: one could be approved and the other not if the former offers enough of a benefit to offset the risk. So, whereas the principle of no or minimal risk sets a fixed maximum for a device's riskiness, taking into account the benefits conferred by use of the device changes what level of risk is considered acceptable. Which approach should be taken when it comes to CEDs?

Whilst we agree that the risks that CEDs pose should be kept as low as reasonably possible without sacrificing benefits, we do not support an approach that allows considerations of risk to trump benefits, as in the ‘no or minimal’ risk approach. We do not think that it would be impossible or inappropriate for regulators to conduct a benefit assessment to help ascertain the maximum level of risk that should be tolerated if a CED is to be placed on the market. This approach would allow that some risks greater than minimal could be offset by benefits conferred by CEDs. As noted above, many of the benefits of cognitive enhancement are similar in nature to medical benefits—improved memory, improved concentration—despite affecting individuals already deemed ‘healthy’ by the medical profession. However, we go on to argue below that this assessment of benefits is unlikely to proceed in the same way as it does for medical devices, and that consideration should be given to the differential value that consumers place on these benefits, consequently erring on the side of consumer choice. Our next task is to suggest how the benefits of CEDs should be assessed and weighed within the framework of the MDD with its emphasis on effectiveness.

#### ‘Benefit’ as a measure of effectiveness

We argue that, whilst equating benefit with effectiveness may be a sound strategy for assessing the marketing and use of neurotechnologies in the clinical context, when CEDs are marketed to competent individuals not considered unwell the MDD should approach this assessment differently. We argue that 1) ‘benefit’ should be understood as something broader than mere effectiveness and 2) the requirement of strong evidence of benefit should (partly as a consequence) be relaxed (see section 4.3). Although (as we note above) it is possible to measure the size of improvements to cognitive function, the value of cognitive enhancement will depend on the circumstances specific to each individual. Improvement of memory for an active researcher will have a different value to improvement of memory for a retired gardener, even though both will have some objective value determinable through measures of effectiveness.

Consequently, we suggest that in relation to CEDs, ‘benefit’ should be understood as an estimation of the technology's propensity to increase wellbeing—that is, roughly, to increase the individual's chances of living a good life.^67^ Crucially, what capacities and traits confer wellbeing will vary depending on the person's goals and values, their nature and their circumstances. This wellbeing-based approach could, in fact, also be used to assess the benefits of treatments. For example, we could assess the benefits of a neurotechnology that is used to alleviate symptoms of Parkinson 's disease by determining the likely effect of the intervention on the wellbeing of the individual.^68^^,^^69^

It could be asked why we allow that benefit should still be understood as effectiveness when assessing medical devices for use in the clinical context, if this concept of increase-to-wellbeing can encompass both effects seen as treatments and effects seen as enhancements? In fact, we are not committed to endorsing the effectiveness-based approach in relation to therapeutic benefits. However it might be argued in favour of that approach that the ‘therapeutic’ effects of the clinical applications are likely to be necessary for leading a good life on most plausible conceptions of such a life. Such applications lead to increment in what Rawls (1971) called ‘primary goods’; goods that enhance an individual's capacity to live a good life, no matter what their conception of the good.^70^ Perhaps, then, a device's degree of effectiveness will be closely related to the extent to which its effects promote wellbeing.

Further, and we expand on this point below, decisions about undergoing an intervention made in the clinical context are importantly different from the decisions made in the non-clinical context due to the particular vulnerabilities present when one's health is in jeopardy. Understanding the size of benefit as the degree of effectiveness in the clinical context serves as a justifiable safeguard.

### Challenge three: setting the regulatory standard (a low-risk exemption for CEDs?)

We suggest that as medical need falls, consumer freedom-to-choose should rise, other things being equal. Whilst the informed consent of patients is routinely obtained before proceeding with any intervention, a patient's decline in health puts her in a vulnerable position where it is likely she will be inclined to accept the treatments on offer. This inclination may be bolstered by the perception that the intervention on offer is ‘endorsed’ by the medical profession, with its authority. This being the case, objective evidence of effectiveness (benefit) must be gathered before offering interventions posing any risks. However, decisions about the purchase and use of enhancement devices are made absent these vulnerabilities, which justifies giving individuals more choice about how to assess the risks and benefits of any particular device in the context of their own values, nature and life circumstances—an assessment they are typically best placed to make for themselves.

To be clear, in advocating consumer freedom-to-choose, we do not propose that devices of all levels of risk are to be approved for placement on the market—this would defeat the purpose of regulation and would be inconsistent with the approach taken to risk both in clinical medicine and in product safety regulation, among other areas. Consumers should be protected from CEDs presenting a risk-benefit profile worse than some agreed threshold, allowing consumers to decide what (small to moderate) risks they wish to take. In defending the position that consumers are best placed to evaluate the risks and benefits of CEDs, we do not wish to suggest that consumers are better placed than experts to determine the nature, size and probability of the effects of CEDs. This is clearly a job for scientists and other experts. Instead, we are suggesting that consumers are best placed to evaluate the impact of these (expert-identified) effects on their own wellbeing. Experts are to assess what the risks are, the consumer how much they matter. Indeed, this is why we later propose a strict requirement that manufacturers include clear, detailed, evidence-based information on the risks of the devices they market. We thus maintain that, whilst there is a good case for imposing strict risk-based restrictions on therapeutic medical devices in order to protect vulnerable patients, for CEDs there may be an argument for placing decisions about the level of acceptable risk primarily in the hands of the consumers who will use them.

Given our preference to promote consumers’ freedom-to-choose, we suggest that consideration should also be given to incorporating a ‘low-risk exemption’, whereby any device that falls under a given level of risk would be approved regardless of whether clinical assessment confirms *any* consistent objective benefits. Devices posing a risk greater than this ‘low-risk’ threshold will have to demonstrate some objective benefits although, as proposed above, given the variation in the value different individuals will place on these benefits and the absence of a straightforward measure of ‘effectiveness’, regulatory assessment should err on the side of allowing consumers to conduct their own evaluations of whether the risks outweigh the benefits. Again, this is not to say that CEDs posing significant risks should always be approved as long as there is at least *some* benefit. Rather, in cases where it is contested whether the benefits make the risks acceptable—where there is room for reasonable disagreement—the device should be approved so that the consumer can adjudicate for herself. Given this room for consumer discretion, we suggest a stringent supplementary requirement for manufacturers to provide transparent, detailed, evidence-based information pertaining to the mechanisms, risks and effects that might be construed as benefits of the devices. Providing such detailed information is currently not compulsory.

It should be noted, however, that the argument for increasing consumer freedom would not apply to CEDs intended for use on children, who are arguably always a vulnerable group. For CEDs developed for children, stringent risk-based restrictions might still be appropriate. Moreover, even CEDs not intended for use in children might in some cases be offered to children. If such devices are freely available, parents could use them on their children without the child's valid consent. So while respect for liberty speaks in favour of liberal regulation of enhancement devices, we propose that criminal sanctions be considered for cases in which untrained adults use CEDs on children without suitable supervision. Similarly to the imposition of sanctions for giving children alcohol, on our proposal (adults’) freedom to purchase and use CEDs is preserved whilst children are protected by placing legal restrictions on the freedom to use CEDs on *them*.

In addition to the risks and benefits likely to affect individual users of CEDs, consideration must be given to the potential size of indirect costs to the healthcare system if faulty devices are used or if devices are misused. This consideration should be weighed against the resources that would be saved if low-risk devices were not subject to ongoing regulation under the MDD.

### Summary of our prescriptive model for risk-benefit assessment of CEDs

Broadly, devices will fall into one of three categories of risk profile: high, moderate or low. The assessment will proceed differently depending on the category.

***Devices with a high risk profile***. There should be some level of risk above which no CED will be approved for sale on the market. Where a device poses such a high risk, any attendant benefits will be irrelevant. Although determining precisely what constitutes a high risk profile will require further discussion, a high-risk device might be one that, for example, is likely to induce seizures.

***Devices with a moderate risk profile***. Where CEDs possess moderate risk profiles, there must be at least some demonstrable benefits of the device to users. The size of these benefits can be measured in a similar way to medical devices: improvements in capacities such as memory or concentration are quantifiable. However, given that people will value these benefits to different degrees, and given the absence of the particular vulnerabilities that attend the medical context, the risk-benefit assessment should err on the side of allowing consumers to decide whether the risks are worth taking. In practical terms, this will mean that the regulatory assessment will not require the objective benefits to clearly outweigh the risks. The exception to this method of assessment is where devices are intended by the manufacturer to be used on children or other vulnerable third parties. In such cases, the objective benefits must justify the risks in the same way as they must for medical devices. Our further proposal is that, although moderate-risk CEDs that are intended for use on vulnerable third-parties might be approved for use by formally-trained practitioners, they should not be approved for sale on the wider market.

***Devices with a low risk profile***. Where the risks posed by a device are low, there need not be any evidence of objective benefit and the device should be excluded from ongoing clinical assessment. Again, the exception to this is where devices are intended by the manufacturer to be used on children or other vulnerable third parties. In such cases, objective benefits must be shown to justify even the low risks in order for the device to be approved for the market.

## POINTS OF COMPARISON WITH IMPLANTABLE OR OTHER INVASIVE PRODUCTS

### Point one: unreasonable broadening of the remit of the MDD

One concern raised by the European Commission when discussing how to extend the MDD to cover some implantable or other invasive products without a medical purpose was to ensure that the remit of the MDD was not unreasonably broadened. If the directive were extended to cover all implantable and invasive products, then things such as earrings and other body piercings would then fall within its remit.^71^ Similarly, if a category of ‘cognition improving’ or ‘brain modifying’ devices were to be added to the definition, it would be very difficult to justify the inclusion of tDCS devices but the exclusion of, for example, educational software. To avoid this unreasonable broadening, either particular mechanisms of action would have to be specified (eg electrical stimulation), or a positive list identifying specific devices would have to be drawn up. For the implantable and other invasive products, the European Commission proposed to solve this by generating a positive list of devices:^72^

With the suggested two-step approach, the incorporation of a general provision regarding implantable or other invasive nonmedical products in the medical device legislation would not have any immediate impact on these products. Only the inclusion in a ‘positive list’ would trigger the application of the legal requirements regarding a given type of products. This would have the advantage that the concrete impacts on specified products could be assessed once a type of product should be added to the positive list.

We believe that given these benefits of a positive list, this approach is preferable to regulating CEDs by reference to their mechanism of action.

### Point two: implications for manufacturers

In relation to implantable and other invasive products, the European Commission also considered the implications for manufacturers, particularly of the more demanding requirements the MDD makes for pre-market clinical assessment. When a manufacturer markets a device for both medical and non-medical purposes, the impact will likely be negligible, as they will already be complying with the requirements of the medical device legislation. However, those that only manufacture devices for nonmedical purposes will begin to be subject to onerous requirements and additional costs. In the case of non-corrective contact lenses:^73^

Manufacturers of only non-corrective contact lenses would have, among others, to draw up a technical documentation (incl. clinical evaluation), be subject to a conformity assessment procedure by a Notified Body and set up a system to respond to incidents (vigilance) which would lead to additional costs. In the case of responsible manufacturers which already today apply an internal quality management system and follow-up of incidents, the additional costs would be limited to the involvement of a Notified Body. Manufacturers which place decorative contact lenses on the market without prior internal quality control and incident follow-up would have to adapt or lose Europe as a market place which would be a desired consequence of this policy option and increase consumer safety.

We suggest that the loss of Europe as a market for manufacturers who do not implement internal quality control and incident follow-up strategies is actually desirable. Similarly, manufacturers making CEDs should have to meet the required standards and incur the costs of doing so in the interests of consumer safety.

However, there may be some concerns about what exactly is required for adequate clinical assessment, especially when devices are in early stages of innovation and have not yet been subject to many clinical trials. In the case of the cosmetic implantable and invasive devices:^74^

The application of the medical device legislation to implantable or other invasive products without a medical purpose may force some products out of the market in case that the manufacturer cannot demonstrate conformity with the essential requirements based on clinical data. In particular, those manufacturers who cannot rely on clinical data obtained for medical devices of the same category would, for ethical reasons, unlikely be allowed to conduct a clinical investigation with a product that does not have a medical purpose. Such effect, however, would ensure that only those non-medical products would be allowed on the EU market for which the manufacturer can prove the same level of safety and performance as for a similar medical device for which the demonstration of the conformity with the essential requirements by means of clinical data is required by law.

Again, to the extent that the devices are already being tested for medical purposes, an adequate assessment of clinical data should be possible. Where devices are being developed purely for enhancement purposes, holding them to these requirements may stifle innovation as ethical approval for clinical investigation may be withheld. Altering the legislation to include CEDs may therefore have ramifications backwards, to the regulation of innovation and clinical research. However, this should not be a concern as, in our view, pre-market assessment should be the same for CEDs as for medical devices if the theoretical risks are similar. Whether the existing research ethics requirements are reasonable is a question that can be posed in relation to both CEDs and medical devices and is beyond the scope of this paper.

## LIMITS TO THE MODEL

Whilst our proposed model would initiate regulation of the market in CEDs, it would not prevent users constructing devices completely from scratch. Further, our proposal has made no recommendations pertaining to the regulation of (mis)use of these devices (other than the suggestion that untrained use on children should attract criminal sanctions). However, whilst the potential misuse of devices would remain a concern even if the devices themselves were regulated, the current lack of regulation is likely to give users the impression that there are no significant risks associated with buying and using CEDs. Further, regulating CEDs may have the effect of encouraging people to purchase a regulated device, rather than build their own. The outcome of our regulatory model would therefore be to filter the most dangerous enhancement technologies out of the market, leaving individuals free to choose which small-to-moderate risks they are willing to take in pursuit of their wellbeing. It also imposes requirements on manufacturers to provide enough detailed, honest information about the product to enable individuals to use the devices in the safest way possible, in full knowledge of all known risks and side effects.

## SUMMARY OF OUR PRESCRIPTIVE MODEL FOR THE REGULATION OF CEDs

Based on the above discussion, we recommend the following for the regulation of CEDs:

- **CEDs should be regulated within the MDD**: the justifications for this are that CEDs have similar mechanisms and risk-profiles to some medical devices and are often essentially the same device; parsimony in legislation is desirable; and the inclusion of some cosmetic implantable and invasive devices sets a precedent for broadening the remit of the directive in this way.
- **A ‘positive list’ of ‘cognition improving or facilitating devices’ should be drawn up**: although this means that the legislation has to react to the emergence of hitherto unregulated devices as they come on to the market, the extension of the directive to all cognition improving or facilitating devices would generate huge difficulties for regulators in keeping the purview of the directive appropriately narrow.
- **The devices that should be included on the initial positive list are**: transcranial electrical stimulation (eg, tDCS, transcranial random noise stimulation, transcranial alternating current stimulation); transcranial magnetic stimulation; neurofeedback equipment.
- **For CEDs presenting a moderate risk profile, benefits should be identified and weighed against risks in a similar (but not identical) way to the assessment made for medical devices**: unlike cosmetic enhancement, improvements elicited by CEDs are more easily quantifiable, and in many cases it may be possible to assess these improvements using standard tests. Assessing the benefits of CEDs in this way gives an estimation comparable to the assessment of the effectiveness of medical devices. However, given that people will *value* these benefits to different degrees, and given the absence of the particular vulnerabilities that attend the medical context, the risk benefit assessment should err on the side of allowing consumers to decide whether the risks are worth taking. In practical terms, this will mean that the regulatory assessment will not require the objective benefits to clearly outweigh the risks.
- **Prohibit CEDs with high risk profiles:** where a device poses significant risks (such as likely seizures) that substantially outweigh its benefits a device should be prohibited from sale on the market.
- **Exempt CEDs with low risk profiles from continued regulatory oversight:** where CEDs are deemed to be low-risk and are unlikely to generate large indirect costs to the healthcare system, there would be a case for exempting them from continued regulatory evaluation, regardless of whether objective benefits have been demonstrated. This promotes consumer choice. Neurofeedback devices would be an example of a low-risk CED unlikely to require ongoing evaluation.
- **Require manufactures to provide consumers with comprehensive, evidence-based information about mechanisms, safe use, risks and benefits:** by making this a stringent requirement for CEDs within the MDD, consumers will be better equipped to make informed decisions about the risks they are willing to take.
- **Limit the low-risk exemption to protect vulnerable parties:** there ought to be an exception to our low-risk exemption proposal when devices are intended for use on/by vulnerable third parties such as children. For such devices, evidence of objective benefit (effectiveness) should be required and weighed against the risks, as for medical devices.
- **Create supplementary criminal sanctions to protect non-competent third parties:** due to the possibility that individuals lacking adequate training could use CEDs that are intended for adults on children or vulnerable adults, we propose that such use should attract criminal sanctions in the same way as supplying children with alcohol attracts criminal sanctions.

## Footnotes

1 British Medical Association Expert Group on Cognitive Enhancements, Boosting your brainpower: ethical aspects of cognitive enhancements, British Medical Association Discussion Paper (2007).

2 Hannah Maslen, Tom Douglas, Roi Cohen Kadosh, Neil Levy & Julian Savulescu, *Do-It-Yourself Brain Stimulation: A Regulatory Model*, JME Online First, published on July 30, 2013 as 10.1136/medethics-2013-101692.

3 Hannah Maslen, Julian Savulescu, Thomas Douglas, Neil Levy & Roi Cohen Kadosh, *Regulation of Devices for Cognitive Enhancement*, *The Lancet* 382, no. 9896 (2013): 938–9.

4 For a more accessible overview of our proposal please see our non-academic Oxford Martin School policy paper aimed at a lay audience: Hannah Maslen, Tom Douglas, Roi Cohen Kadosh, Neil Levy & Julian Savulescu, *The Regulation of Cognitive Enhancement Devices*. The present paper engages in far more depth with the relevant legislation and the challenges and opportunities it presents for implementing our model.

5 Editorial, *Brain Blast: DIY Attempts at Electrical Brain Stimulation to Improve Cognition Are to Get Easier*, Nature 498, 271–2 (2013).

6 Examples include: http://www.foc.us/; http://www.biocurrentkit.com/; http://www.trans-cranial.com/ (accessed 3 October 2013).

7 The new foc.us device was listed as sold out when the website was accessed on the 3 October 2013.

8 http://www.york-biofeedback.co.uk/neurofeedback/tdcs.aspx (accessed 3 October 2013).

9 Charlotte J. Stagg, Michael A. Nitsche, *Physiological Basis of Transcranial Direct Current Stimulation*, 17 The Neuroscientist 37–53 (2011).

10
Lilly Knechtel, Renate Thienel & Ulrich Schall, *Transcranial Direct Current Stimulation: Neurophysiology and Clinical Applications*, 3 Neuropsychiatry 89–96 (2013).

11 Roi Cohen Kadosh, ‘*Using Transcranial Electrical Stimulation to Enhance Cognitive Functions in the Typical and Atypical Brain*’, 4 Translational Neurosc. 20–33 (2013).

12 For an overview of the risks and safety concerns outlined here, see Nicholas S. Fitz, Peter B. Reine, *The Challenge of Crafting Policy for Do-It-Yourself Brain Stimulation*, JME Online First, published on June 3, 2013 as 10.1136/medethics-2013-101458.

13 Andre Russowsky Brunoni, Michael A. Nitsche, Nadia Bolognini, et al., *Clinical Research with Transcranial Direct Current Stimulation (tDCS): Challenges and Future Directions*, 5 Brain Stimulation 175–95 (2012).

14 For a pilot study investigating the significance of handedness for the effects of tDCS, see Sebastian Schade, Vera Moliadze, Walter Paulus, Andrea Antal, *Modulating Neuronal Excitability in the Motor Cortex with tDCS Shows Moderate Hemispheric Asymmetry Due to Subjects’ Handedness: A Pilot Study*, 30 Restor. Neurol. Neuros. 191–8 (2012).

15 Stagg and Nitsche, *supra* note 9, at 43.

16 Michael A. Nitsche, Leonardo G. Cohen, Eric M. Wassermann, et al., ‘*Transcranial Direct Current Stimulation: State of The Art*’, 1 Brain Stimulation 206–23 (2008).

17 As above, many of the safety concerns outlined here are also identified by Fitz and Reiner, *supra* note 12.

18 Brunoni et al., *supra* note 13, at 183.

19 Id.

20 Roi Cohen Kadosh, Sonja Soskic, Teresa Iuculano, et al., *Modulating Neuronal Activity Produces Specific and Long-Lasting Changes in Numerical Competence*, 20 Curr. Biol. 2016–20 (2010).

21 Teresa Iuculano & Roi Cohen Kadosh, *The Mental Cost of Cognitive Enhancement*, 33 J. Neurosc. 4482–6 (2013).

22 Megan Spencer-Smith, Vicki Anderson, *Healthy and Abnormal Development of the Prefrontal Cortex*, 12 Dev. Neurorehabil. 279–97 (2009).

23 Roi Cohen Kadosh, Neil Levy, Jacinta O'Shea, et al., *The Neuroethics of Non-Invasive Brain Stimulation*, 22 Curr. Biol. R108–11 (2012).

24 D. Corydon Hammond, *What is Neurofeedback? An Update*’, 15 J. Neurotherapy 305–36 (2011).

25 Id.

26 For example: http://store.brainmaster.com/; http://brain-trainer.com/; http://www.smartbraintech.com/ (accessed 3 October 2013).

27 http://www.york-biofeedback.co.uk/neurofeedback/neurofeedback_york.aspx (accessed 3 October 2013).

28 Hammond, *supra* note 24.

29 Gruzelier J. H., Foks M., Steffert T., Chen M. L. & Ros T., *Beneficial Outcome from EEG-Neurofeedback on Creative Music Performance, Attention and Well-Being in School Children*, 95 Biological Psychology 86–95 (2014).

30 Joshua Raymond, Imran Sajid, Lesley A. Parkinson & John H. Gruzelier, *Biofeedback and Dance Performance: A Preliminary Investigation*, 30 J. Appl. Psychophysiol. Biofeedback 65–73 (2005).

31 John Gruzelier, Atsuko Inoue, Roger Smart, et al., *Acting Performance and Flow State Enhanced with Sensory-Motor Rhythm Neurofeedback Comparing Ecologically Valid Immersive VR and Training Screen Scenarios*, 480 Neurosc. Lett. 112–6 (2010).

32 Corydon Hammond & Lynda Kirk, *First, Do No Harm: Adverse Effects and the Need for Practice Standards in Neurofeedback*, 12 J. Neurotherapy 79–88 (2008).

33 Id.

34 Thomas V. Matthews, *Neurofeedback Overtraining and the Vulnerable Patient*, 11 J. Neurotherapy 63–6 (2007).

35 Hammond, *supra* note 24; Corydon D. Hammond, *The Need for Individualization in Neurofeedback: Heterogeneity in QEEG Patterns Associated with Diagnoses and Symptoms*, 35 Appl. Psychophysiol. Biofeedback 31–6 (2010).

36 Ghaziri J., Tucholka A., Larue V., et al., *Neurofeedback Training Induces Changes in White and Gray Matter*, 44 Clinical EEG Neurosc. 265–72 (2013).

37 Directive 2001/95/EC.

38 Directive 93/42/EEC.

39 *Id.* at 5–6.

40 http://eur-lex.europa.eu/LexUriServ/LexUriServ.do?uri=SWD:2012:0273%2852%29:FIN:EN:PDF, Commission staff working document: Impact assessment on the revision of the regulatory framework for medical devices, European Commission at 6.

41 Id.

42 See: http://epochproject.com/ (accessed 20 March 2013).

43 Report from a Joint Workshop Hosted by The Academy of Medical Sciences, The British Academy, The Royal Academy of Engineering and The Royal Society (2012), Human Enhancement and the Future of Work, The Academy of Medical Sciences.

44 Simon M. Outram & Eric Racine, *Examining Reports and Policies on Cognitive Enhancement: Approaches, Rationale, and Recommendations*, 18 Accountability in Research: Policies and Quality Assurance 323–41 (2011).

45 British Medical Association, *supra* note 1.

46 *Id.* at 1.

47 The Academy of Medical Sciences, *supra* note 43, at 51.

48 Fitz and Reiner, *supra* note 12.

49 Nuffield Council on Bioethics Report on Novel neurotechnologies: intervening in the brain (2013), Nuffield Council on Bioethics.

50 Knechtel et al., *supra* note 10.

51 See, for example, Leon R. Kass, *Ageless Bodies, Happy Souls: Biotechnology and the Pursuit of Perfection*, 1 The New Atlantis 9–28 (2003); President's Council on Bioethics, Beyond Therapy: Biotechnology and the Pursuit of Happiness (2003).

52 See, for example, J. Harris, Enhancing evolution: the ethical case for making better people (2007); A. Bunchanan, Better than Human: The Promise and Perils of Enhancing Ourselves (2011).

53 Cohen Kadosh et al., *supra* note 23.

54 British Medical Association, *supra* note 1, at 34.

55 *Id.* at 34.

56 Cohen Kadosh, *supra* note 11.

57 The devices identified for inclusion are: contact lenses; implants for modification or fixation of body parts; facial or other dermal or mucous membrane fillers; equipment for liposuction; invasive laser equipment intended to be used on the human body; and intense pulsed light equipment.

58 See, for example: E. T. Juengst, *What Does “Enhancement” Mean?*, in Enhancing Human Traits: Ethical and Social Implications (E. Parens ed., 1998); J. Savulescu, *Genetic Interventions and the Ethics of Enhancement of Human Beings*, in The Oxford Handbook on Bioethics (B. Steinbock ed., 2006).

59 European Commission, *supra* note 40, at 17.

60 European Commission's documents on Medical Devices: Guidance document - Guidelines relating to the application of: The council directive 90/385/EEC on active implantable medical devices the council directive 93/42/EEC on medical devices (1994), Medical Devices: Guidance Document, European Commission, p. 3, http://ec.europa.eu/health/medical-devices/files/meddev/2_1-1__04-1994_en.pdf

61 Note, however, that there is at least conceptual space to make a similar point in relation to therapeutic medical devices. A device might be 90% effective at curing headaches, but how beneficial this is will depend on how bad one finds headaches, what activities one's headaches frustrate and so on.

62 European Commission's Document on Proposal for a Regulation of the European parliament and of the council on medical devices, and amending Directive 2001/83/EC, Regulation (EC) No 178/2002 and Regulation (EC) No 1223/2009 (2012), European Commission, p. 101, http://ec.europa.eu/health/medical-devices/files/revision_docs/proposal_2012_542_en.pdf

63 European Commission's Document on Guidelines on Medical Devices: Clinical Evaluation: A Guide For Manufacturers And Notified Bodies (2009), European Commission, p. 16, http://ec.europa.eu/health/medical-devices/files/meddev/2_7_1rev_3_en.pdf.

64 European Commission, *supra* note 62, at 102.

65 MHRA's Document on The revision of European legislation on medical devices, MHRA 10 (2012). http://www.mhra.gov.uk/home/groups/comms-ic/documents/publication/con205362.pdf.

66 SPECTARIS is the German trade association representing the National and International Companies of Contact Lens (and Lens Care) Manufacturers),*Response to Public Consultation Recast of the Medical Devices Directives*, Unit ENTR F/3, Cosmetics and Medical Devices, SPECTARIS.

67 See J. Savulescu, A. Sandburg and G. Kahane, *Well-being and Enhancement*, in Enhancing Human Capacities (J. Savulescu, R. ter Meulen, G. Kahane eds., 2011).

68 G. Kahane and J. Savulescu, *The Welfarist Account of Disability*, in Disability and Disadvantage 14–53 (K. Brownlee and A. Cureton eds., 2009).

69 To a certain extent, healthcare resource allocation agencies such as NICE adopt this approach to ‘effectiveness’ by taking improvements (or impairments) to the quality of a person's into account. The clinical effectiveness of different drugs is measured using the quality-adjusted life years measurement (the ‘QALY’). See: http://www.nice.org.uk/newsroom/features/measuringeffectivenessandcosteffectivenesstheqaly.jsp (accessed 18 October 2013).

70 J. Rawls, A Theory of Justice (1971, revised edition 1999).

71 European Commission, *supra* note 40, at 11.

72 *Id.* at 15.

73 *Id.* at 16.

74 *Id.* at 16–7.

